# Sex-Specific Anti-Inflammatory Effects of a Ketogenic Diet in a Mouse Model of Allergic Airway Inflammation

**DOI:** 10.3390/ijms26073046

**Published:** 2025-03-26

**Authors:** Carolyn D. Ekpruke, Omar Borges-Sosa, Christiane A. Hassel, Dustin Rousselle, Lyidia Dinwiddie, Maksat Babayev, Ahmed Bakare, Patricia Silveyra

**Affiliations:** 1 Department of Environmental and Occupational Health, School of Public Health, Indiana University, Bloomington, IN 47405, USA; cekpruke@iu.edu (C.D.E.); oborges@iu.edu (O.B.-S.); drousse@iu.edu (D.R.); didinwi@iu.edu (L.D.); mbabayev@iu.edu (M.B.); 2 Flow Cytometry Core Facility, Indiana University, Bloomington, IN 47405, USA; chassel@iu.edu; 3 School of Medicine, Johns Hopkins University, Baltimore, MD 21287, USA; wlabwise@gmail.com; 4 School of Medicine, Indiana University, Indianapolis, IN 46202, USA

**Keywords:** ketogenic diet, immune cells, allergic airway inflammation, sex differences, allergic asthma

## Abstract

Asthma, a chronic inflammatory airway disease, leads to airflow obstruction and exhibits sex differences in prevalence and severity. Immunomodulatory diets, such as the ketogenic diet (high fat, low carbohydrate, moderate protein), may offer complementary benefits in managing airway inflammation. While anti-inflammatory effects of ketogenic diets are documented in cardiovascular diseases, their impact on asthma, especially regarding sex-specific differences, remains unexplored. Few studies on diet and asthma have considered sex as a biological factor. To test the hypothesis that a ketogenic diet affects airway inflammation in a sex-specific manner, we used a mouse allergic airway inflammation model. Male and female C57BL/6J mice (3–4 weeks old, *n* = 5–6/group) were fed a ketogenic diet or normal chow for 12 weeks. From weeks 7 to 12, mice were challenged intranasally with house dust mite allergens (HDM) 5 days/week to induce airway inflammation. Lung tissue was analyzed 72 h post-exposure using flow cytometry to assess immune cell populations, and data were analyzed with two-way ANOVA. The ketogenic diet increased body weight in allergen-exposed mice, with a greater effect in males than females (*p* = 0.0512). Significant sex–diet interactions were noted for alveolar macrophages, CD103^+^, CD11B^+^, and plasmacytoid dendritic cells (*p* < 0.05). Eosinophil reductions were observed in males but not females on the ketogenic diet. The diet also increased NKT cells and decreased NK cells in males but not females (*p* < 0.001). These findings highlight sex-specific effects of ketogenic diets on lung immune responses, with stronger impacts in males.

## 1. Introduction

Asthma is a common heterogeneous disease, usually associated with chronic airway inflammation, leading to airflow obstruction. According to recent health surveys, asthma affects over 339 million people worldwide and an estimated 25 million in the United States. Notably, there are significant sex differences in the incidence and prevalence of asthma across the lifespan [1,2], with adult women experiencing higher prevalence and severity than men. This difference is influenced by factors such as airway size, immune response, and hormonal effects, particularly estrogen [1]. Diet is a major environmental factor that can alter normal organ development and the development of lung diseases such as asthma, as it is a known disease modifier. Diet and asthma have been associated previously, but the strength of this association remains unclear [3,4]. Diet is an important immunomodulator and intervention in the treatment of respiratory dysfunction. For example, diet has been linked to the severity of asthma symptoms in children [5], and improvement of diet quality can control asthma outcomes [6]. However, animals on high sucrose diets showed an increase in airway hyperresponsiveness [7]. Similarly, an increase in airway inflammation was observed in patients after consuming a high-fat diet [8,9]. On the other hand, administering oils with linolenic and eicosatetraenoic acids significantly reduces inflammatory mediators associated with asthma [10].

Ketogenic diets are high in fats and low in carbohydrates with moderate protein. Prior studies have shown that ketogenic diets can be used to treat drug-refractory epilepsy and have an anti-inflammatory effect [11]. The low carbohydrate content of the ketogenic diet is responsible for metabolic induction, making the liver produce more ketone bodies from fatty acids. In turn, these ketone bodies enter the circulation and are used as fuel in body tissues, where they release fewer free radicals than carbohydrates [12]. Because asthma has its origin in damaging free radical reactions [13], the antioxidant and anti-inflammatory properties of ketone bodies could be beneficial for its treatment and management, as evidenced in animal models [14,15,16,17]. The ketogenic diet can also decrease the respiratory exchange ratio in healthy individuals [18] and improve inflammation and lung function in chronic obstructive pulmonary disease (COPD) [19].

Recently, the ketogenic diet has been used as a weight loss intervention, even within a short term [20]. In patients with diabetes, Walton et al. reported lower body weight, blood pressure, blood lipids, and blood glucose levels after 90 days of a ketogenic diet [21]. Because obesity has been suggested as a risk factor for asthma and its exacerbations [22], the effects of various diets on asthma patients have been studied [23]. While a positive association has been identified between reduction in weight and improved asthma symptoms [23], it remains unclear whether the improvement of asthma symptoms observed among obese patients after a ketogenic diet administration is due to the effect of the diet or weight reduction [24]. Additionally, sex has not been considered a biological factor in the impact of a ketogenic diet on chronic diseases [25].

Regarding the lungs, a recent study using two obese mouse models (one fed a high-fat diet and one genetically obese) found a decrease in airway hyperresponsiveness after feeding a ketogenic diet for three weeks [26]. Changes in pro-inflammatory gene expression and microbiome composition were also found [26]. While some of their experiments included males and females, others were conducted only in males, and results were not stratified by sex. Based on our prior work demonstrating sex differences in lung inflammation, airway hyperresponsiveness, and microbiome composition in an asthma model [27,28,29], and the known sex-specific association of asthma and obesity [30,31], we hypothesized that the ketogenic diet would display a sex-specific anti-inflammatory property. Thus, this study investigated sex differences in the impact of a ketogenic diet on airway inflammatory cells in allergen-challenged mice. 

## 2. Results

### 2.1. Impact of Ketogenic Diet on Allergen-Induced Mice

#### 2.1.1. A Ketogenic Diet Induced a Significant Weight Gain in Allergen-Induced Male but Not Female Mice

**Figure 1:**
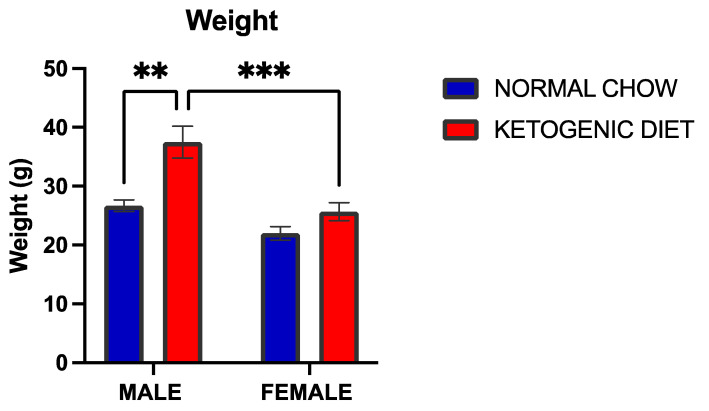
Weight changes in male and female mice (*n* = 5–6 per group) fed a ketogenic diet or normal mice chow and exposed to HDM for 12 weeks from the age of 3–4 weeks old. Results shown as bar charts depict the average weight in the different groups. Error bars denote SEM. ** *p*  ≤  0.01, *** *p*  ≤  0.001.

An increase in weight was observed in allergen-induced males (*p* = 0.0004) but not in females maintained on a ketogenic diet compared to the respective control mice (Figure 1). However, the interaction between diet and sex was not statistically significant (*p* = 0.0512), although there were marked sex differences in the weight of male and female mice in the ketogenic diet (*p* = 0.0001).

#### 2.1.2. Alveolar Macrophages Were Elevated in Allergen-Exposed Mice Fed a Ketogenic Diet

**Figure 2:**
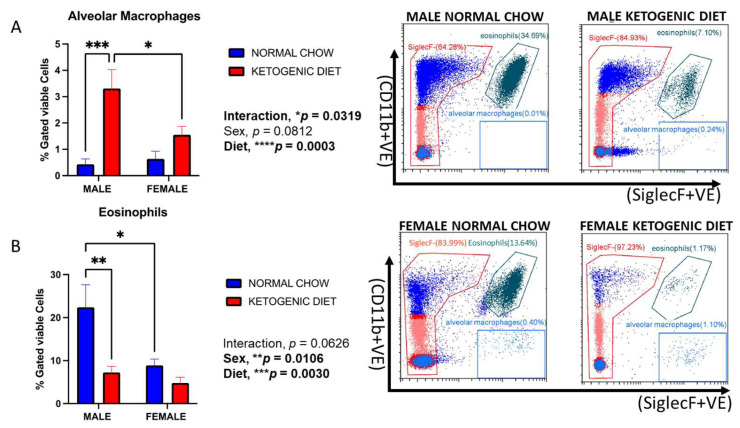
The percentages of alveolar macrophages (**A**) and eosinophils (**B**) in the lungs of allergen-induced male and female mice fed a ketogenic diet or the normal mice chow from 3–4 weeks old for 12 weeks (*n* = 5–6/group). The cells were stained with antibodies and subjected to flow cytometry to determine cell-type percentages, as shown in Materials and Methods. Results shown as bar charts and flow cytometry plots depict average cell percentages (percent of live cells). Error bars denote SEM. * *p*  ≤  0.05, ** *p*  ≤  0.01,*** *p*  ≤  0.001 and **** *p*  ≤  0.0001.

Lung tissue alveolar macrophages were elevated in allergen-induced male and female mice maintained on a ketogenic diet compared to their counterparts fed normal mice chow (Figure 2A). Two-way ANOVA identified a significant interaction of sex and diet (*p* = 0.0319), where the effect was higher in males than in females.

#### 2.1.3. Eosinophils Were Reduced in Allergen-Induced Male Mice Fed a Ketogenic Diet

Lung eosinophils displayed sex differences in animals fed the normal chow, with males higher than females (*p* = 0.0106) (Figure 2B). In contrast, eosinophils were significantly decreased in the lungs of male mice fed a ketogenic diet compared to those fed with the normal mice chow (*p* = 0.003) (Figure 2B). While a reduction trend was also observed for female mice, the trend was not statistically significant. The interaction of sex and diet was also not significant.

#### 2.1.4. Sex Differences in CD8^+^ but Not CD4^+^ Lung T-Cells in Allergen-Induced Mice

**Figure 3:**
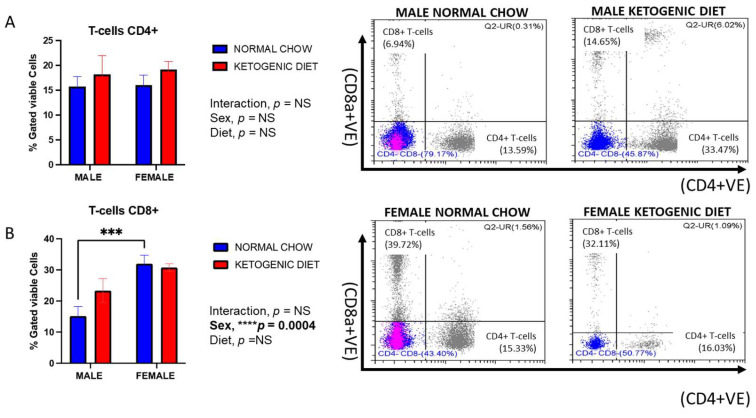
The percentages of CD4^+^ T-cells (**A**) and CD8^+^ T-cells (**B**) in the lungs of allergen-induced male and female mice fed a ketogenic diet or the normal mice chow from 3-4 weeks old for 12 weeks (*n* = 5–6/group). The cells were stained with antibodies and subjected to flow cytometry to determine cell-type percentages, as shown in Materials and Methods. Results shown as bar charts and flow cytometry plots depict average cell percentages (percent of live cells). Error bars denote SEM. *** *p*  ≤  0.001 and **** *p*  ≤  0.0001.

While no sex differences or effects of diet were observed for lung CD4^+^ T-cells (Figure 3A), CD8^+^ T-cells displayed higher counts in female than male mice fed a normal chow (*p* = 0.0004), but not a ketogenic diet (Figure 3B). A trend for higher counts of CD8^+^ T-cells was observed in males fed a ketogenic diet vs. normal chow, but neither this difference nor the interaction of sex and diet were significantly different (Figure 3B).

#### 2.1.5. Sex Differences in Plasmacytoid Dendritic Cells in Response to a Ketogenic Diet and After Allergen Exposure

**Figure 4:**
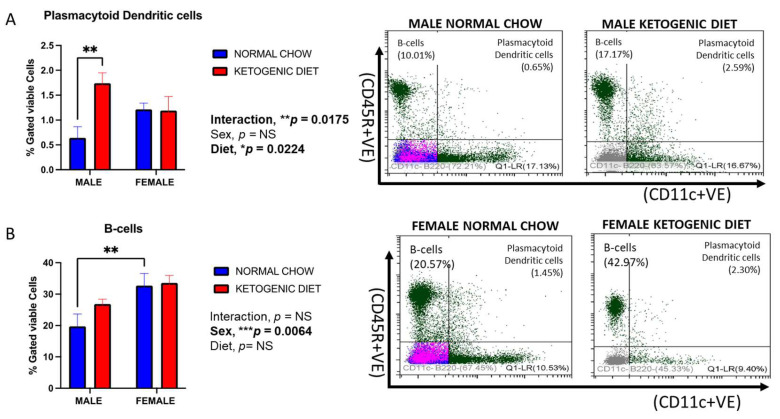
The percentages of plasmacytoid dendritic cells (**A**) and B-cells (**B**) in the lungs of allergen-induced male and female mice fed a ketogenic diet or the normal mice chow from 3–4 weeks old for 12 weeks (*n* = 5–6/group). The cells were stained with antibodies and subjected to flow cytometry to determine cell-type percentages, as shown in Materials and Methods. Results shown as bar charts and flow cytometry plots depict average cell percentages (percent of live cells). Error bars denote SEM. * *p*  ≤  0.05, ** *p*  ≤  0.01, and *** *p*  ≤  0.001.

The percentage of lung plasmacytoid dendritic cells was higher in males fed a ketogenic diet than those on a normal diet (*p* = 0.0224) (Figure 4A). Interestingly, these cells were higher in females than males in the control group and higher in males than females in the ketogenic diet group, but these trends were not significant. However, the interaction of sex and diet was statistically significant (*p* = 0.0175).

#### 2.1.6. Sex Differences in B-Cells in Response to Ketogenic Diet After Allergen Exposure

The percentage of lung B-cells was significantly higher in females than in males fed normal mice chow (*p* = 0.0064) but not a ketogenic diet (Figure 4B). In addition, these cells appear to be elevated in males, but not females, fed a ketogenic diet compared to those fed the normal mice chow, but the trend was not statistically significant. The effect of diet alone, or the interaction between the two factors, was not statistically significant (Figure 4B).

#### 2.1.7. Sex Differences in CD103^+^ and CD11b^+^ Dendritic Cells in Allergen-Induced Mice Fed a Ketogenic Diet

**Figure 5:**
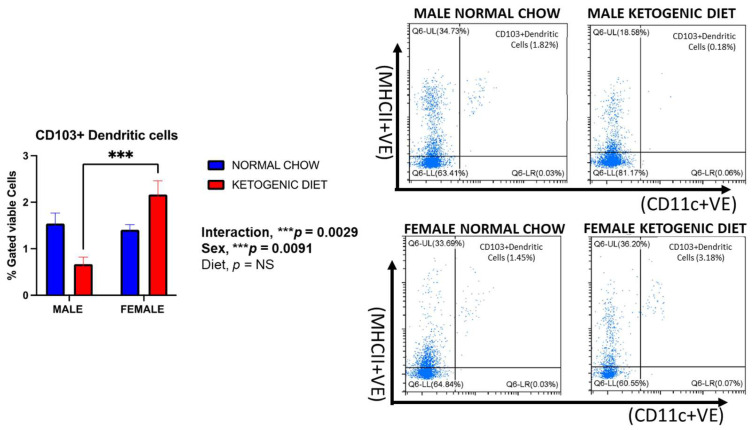
The percentage of CD103^+^ Dendritic cells in the lungs of allergen-induced male and female mice fed a ketogenic diet or the normal mice chow from 3‒4 weeks old for 12 weeks (*n* = 5–6/group). The cells were stained with antibodies and subjected to flow cytometry to determine cell-type percentages, as shown in Materials and Methods. Results shown as bar charts and flow cytometry plots depict average cell percentages (percent of live cells). Error bars denote SEM. *** *p*  ≤  0.001.

**Figure 6:**
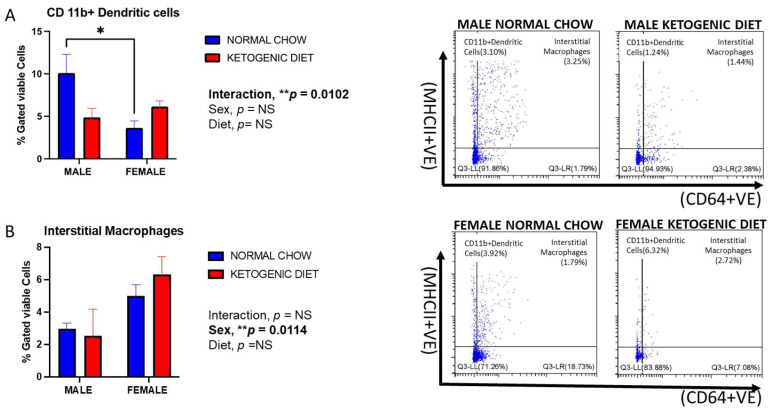
The percentages of CD11b^+^ dendritic cells (**A**) and interstitial macrophages (**B**) in the lungs of allergen-induced male and female mice fed a ketogenic diet or the normal mice chow from 3–4 weeks old for 12 weeks (*n* = 5–6/group). The cells were stained with antibodies and subjected to flow cytometry to determine cell-type percentages, as shown in Materials and Methods. Results shown as bar charts and flow cytometry plots depict average cell percentages (percent of live cells). Error bars denote SEM. * *p*  ≤  0.05 and ** *p*  ≤  0.01.

Lung CD103^+^ dendritic cells were reduced in male mice fed a ketogenic diet compared to those maintained on normal mice chow, although this effect was not statistically significant (Figure 5). Interestingly, this observation was reversed in females, where the percentage of cells was increased in the group fed a ketogenic diet compared to the normal chow. However, there was a marked sex difference, with females displaying higher CD103^+^ dendritic cells than males in the ketogenic diet group (*p* = 0.0091) (Figure 5). While not statistically significant, this trend was also observed for lung CD11b^+^ dendritic cells in mice fed the ketogenic diet (Figure 6A). In contrast, CD11b^+^ dendritic cells were significantly higher in males than females fed the normal chow, while no differences were observed in the ketogenic diet group (Figure 6A). The interaction of sex and diet was statistically significant for CD103^+^ dendritic cells (*p* = 0.0029) and CD11b^+^ dendritic cells (*p* = 0.012).

#### 2.1.8. Sex Differences and Ketogenic Diet Effects on Lung Interstitial Alveolar Macrophages, Undifferentiated Monocytes, and Neutrophils in Allergen-Induced Mice

**Figure 7:**
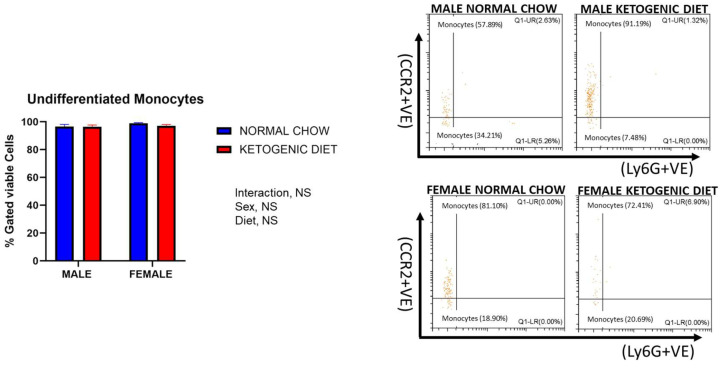
The percentages of undifferentiated monocytes in the lungs of allergen-induced male and female mice fed a ketogenic diet or the normal mice chow from 3–4 weeks old for 12 weeks (*n* = 5–6/group). The cells were stained with antibodies and subjected to flow cytometry to determine cell-type percentages, as shown in Materials and Methods. Results shown as bar charts and flow cytometry plots depict average cell percentages (percent of live cells). Error bars denote SEM. There was no statistically significant difference at *p* < 0.05 in the percentage of those cells in the control and experimental groups.

**Figure 8:**
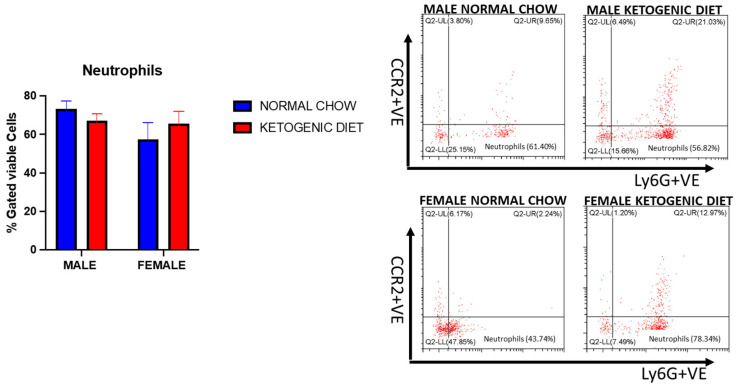
The percentages of neutrophils in the lungs of allergen-induced male and female mice fed a ketogenic diet or the normal mice chow from 3–4 weeks old for 12 weeks (*n* = 5–6/group). The cells were stained with antibodies and subjected to flow cytometry to determine cell-type percentages, as shown in Materials and Methods. Results shown as bar charts and flow cytometry plots depict average cell percentages (percent of live cells). Error bars denote SEM. There was no statistically significant difference at *p* < 0.05 in the percentage of those cells in the control and experimental groups.

Lung interstitial alveolar macrophages were elevated in female mice fed a ketogenic or normal diet compared to males (*p* = 0.0114). Still, the interaction of sex and diet was not statistically significant (Figure 6B). While no statistically significant effects of diet were observed, these cells were generally higher in females fed a ketogenic diet when compared to females fed a normal mouse chow (Figure 6B). On the other hand, we observed no differences in lung undifferentiated monocytes or neutrophils in either males or females fed a normal or ketogenic diet (Figure 7 and Figure 8).

#### 2.1.9. Natural Killer (NK) Cells and Natural Killer T-Cells (NKT) Response in Allergen-Induced Mice Fed a Ketogenic Diet

**Figure 9:**
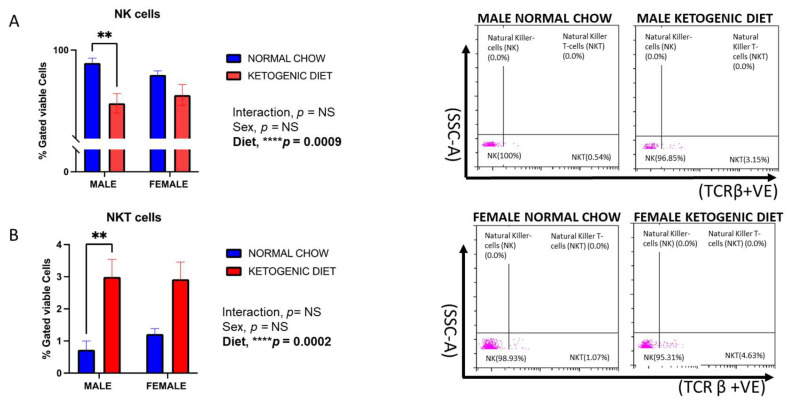
The percentages of natural killer cells (**A**) and natural killer T-cells (**B**) in the lungs of allergen-induced male and female mice fed a ketogenic diet or the normal mice chow from 3–4 weeks old for 12 weeks (*n* = 5–6/group). The cells were stained with antibodies and subjected to flow cytometry to determine cell-type percentages, as shown in Materials and Methods. Results shown as bar charts and flow cytometry plots depict average cell percentages (percent of live cells). Error bars denote SEM. Error bars denote SEM. ** *p*  ≤  0.01 and **** *p*  ≤  0.0001.

We observed a decrease in the percentage of NK cells in mice fed a ketogenic diet compared to those fed a normal chow (Figure 9A). While the interaction of sex and diet was not statistically significant, and there was no effect of sex, the diet effect was only significant in male mice (*p* = 0.0009) (Figure 9A). On the contrary, the lung NKT cells were elevated in male and female mice fed a ketogenic diet compared to their respective control diet groups. Still, this effect was only significant in the male group (*p* = 0.0002) (Figure 9B). The interaction of sex and diet was also not statistically significant for NKT cells.

## 3. Discussion

In this study, we assessed the impact of a ketogenic diet on lung allergic inflammation through lung immune cell profiles in male and female mice. The mice used for this study were maintained on a ketogenic diet or normal mice chow for 12 weeks and exposed to house dust mites intranasally for 5 weeks to induce allergic airway inflammation, a model we have used previously [27,28,29]. We identified sex differences and the interaction of sex and diet for multiple cell types, indicating a sex-specific effect of this diet on allergic inflammation.

The ketogenic diet used consisted of a very high fat content (89%), a very low amount of carbohydrates (1%), and a low amount of protein (10%). The high-fat content was expected to induce the production of ketosis even without fasting, which has been shown to cause a special metabolic condition in mice that results in body weight loss similar to that caused by caloric restriction [32]. We observed a significant increase in the weight of males fed a ketogenic diet but not in the females when compared to the control group, similar to the results reported by other researchers using a live tumor model [33]. Other clinical and animal studies have reported a reduction in body weight when the ketogenic diet was fed to an obese person or animal, and male but not female rats lost bone volume after being fed a ketogenic diet for 12 weeks [32,34,35,36,37]. However, reduced physical activity was reported in mice maintained on a ketogenic diet [38], which may be attributed to the increased body weight observed in the male mice in this study [38]. The weight gain observed in the ketogenic diet groups contrasts with previous studies reporting weight loss or maintenance. This may be due to differences in macronutrient composition, caloric intake, metabolic adaptations, or species-specific responses. Additionally, sex differences, intervention duration, and baseline metabolism could have influenced outcomes. Beyond metabolic effects, diet composition itself may modulate immune function, as high fat intake and carbohydrate deficiency can impact inflammation, cytokine production, and gut microbiota. These findings suggest both metabolic and nutrient-driven immune modulation, warranting further investigation.

Our study revealed that a 12-week dietary intervention can impact the immune cell population in the lungs of an allergen-induced airway inflammation mice model in a sex-dependent manner. The duration and objectives of research influence the method used to analyze the immune cell population, hence the variation seen in different studies [39,40,41,42]. Especially in neuroinflammation, studies have reported a reduction in the production of cytokines, such as IL-1β, IL-6, TNF-α, IL-12, and IL-17, and chemokines, such as IFN-Ɣ, MCP-1, MIP-1a, and MIP-1b [43,44] in allergic asthma. Most studies investigating changes in immune cells used peripheral blood [45,46,47,48], while others used bone marrow and spleen [49]. Previous studies revealed that dietary ketones improved asthma symptoms without necessarily reducing body weight [26]; this agrees with what was observed in our study. While our findings highlight the important role of diet in reshaping the immune system, whether all the tissues in the body respond in the same manner to a ketogenic diet remains unclear. For instance, in our study, we observed an increase in CD4^+^ T-cells and NKT cells in both male and female mice fed a ketogenic diet, and an increase in CD8+ T-cells only in males. Similarly, the work of Goldberg et al. showed the same trend, but in a different disease model, the respiratory influenza virus infection [50,51].

Although there are no recent studies about the effect of ketosis on the immune cell population in the lungs in allergic airway inflammation, older studies have demonstrated that phagocytic activity of neutrophils in the presence of a ketone body, acetoacetate [52,53]. Interestingly, in this current study, neutrophils were not different across groups. The Goldberg et al. study not only reported that long-term ingestion of a ketogenic diet caused obesity in mice but also resulted in an increase in macrophages and decreased γδT-cells in adipose tissue [51]. Even though we observed an increase in lung alveolar macrophages in male and female mice fed a ketogenic diet, lung interstitial macrophages showed no changes. A ketogenic diet is known to elicit responses associated with adaptive immunity links and not innate immunity [54,55]. In our study, this was evident by the significant increase in T- and B-cells, especially in the male mice. However, the changes in alveolar macrophages and eosinophils observed in our mice indicate that the diet can also influence innate immunity allergic responses.

Other cells of interest are dendritic cells, as studies have suggested that these could play a role in the pathogenesis of allergic airway diseases like asthma [56,57,58,59,60] and contribute to eosinophilic inflammation [61]. Geissmann et al. classified dendritic cells into different subsets depending on the surface marker expression or development [62]. These subsets included the pre-conventional dendritic cells, which express either CD103 or CD11b integrins, and the plasmacytoid dendritic cells [44,63], which exert different functions in response to viral and allergic responses [55,64,65,66]. Interestingly, the plasmacytoid dendritic cells are involved in the induction of Treg cells to control airway inflammation [67]. Here, we tested the different subtypes, including CD103^+^, CD11b^+^, and plasmacytoid dendritic cells in response to the diet and allergen challenge. Interestingly, the CD103^+^ and CD11b^+^ dendritic cells displayed differential phenotypes but an interaction of sex and diet. This may represent a novel mechanism through which a ketogenic diet relieves eosinophilic airway inflammation, as eosinophils were observed to be reduced in the group fed a ketogenic diet compared to the control, displayed higher levels in males than females, and are the primary phenotype induced by intranasal HDM instillation [27,28,29]. In contrast, plasmacytoid dendritic cells are known to reduce inflammation when present in large numbers by depleting eosinophils through the production of interferon [43,44] and were increased in males fed a ketogenic diet in our model.

Our findings revealed a significant reduction in katural killer cells (NKs) and increase in natural killer T-cells (NKTs) in the males but not in the female mice maintained on ketogenic diet when compared to those on the normal mice chow. In both human [68,69] and animal studies [70], natural killer cells are known to increase in allergic airway inflammation, they aggravate inflammation by producing type 2 cytokines and inducing eosinophil migration [71,72]. Little is known about the specific role of NKT cells in allergic airway inflammation, although they have been seen to increase in allergic inflammation [73]. However, Hall and Agrawal [74], suggested that NKT cells are emerging mediators that could be a novel therapeutic strategy to improve immunotherapies coupled with their ability to participate in both innate and adaptive immunity.

We observed a significant interaction between sex and diet in multiple immune lung cell profiles in allergen-induced mice. These included alveolar macrophages and dendritic cells (plasmacytoid, CD11b^+,^ and CD103^+^). The effect of sex alone was also significant in the responses of eosinophils, CD8^+^ T-cells, B-cells, and interstitial macrophages. The independent effect of diet was significant in the responses of eosinophils, NK cells, and NKT cells. However, the neither the interaction between sex and diet nor their independent effects were significant in the responses of undifferentiated monocytes, neutrophils, or CD4^+^ T-cells.

The feasibility of humans following a ketogenic diet for managing asthma remains a subject of ongoing investigation. While preclinical studies suggest potential anti-inflammatory benefits, long-term adherence to the diet can be challenging due to its restrictive nature and potential adverse effects, including bone health deterioration, nutritional deficiencies, and metabolic imbalances [75]. Given these concerns, a ketogenic diet is not recommended for treating acute asthma attacks, as standard pharmacological interventions such as bronchodilators and corticosteroids remain the most effective and evidence-based approaches. Further research is necessary to evaluate the long-term safety, effectiveness, and practicality of implementing a ketogenic diet in asthma management.

One limitation of this study was that we did not measure the level of ketone bodies in the animals, and the weight of the animals was not monitored weekly. However, studies have reported an increase in ketone bodies in animals fed a ketogenic diet [76,77]. A second limitation is that we only fed the diets to mice undergoing the allergen challenge, which limits our interpretation of the findings in the context of allergic responses. Additionally, while food intake was not measured in this study, it is strongly recommended as an important factor to consider in future research. However, this study provides a sex-specific mechanistic impact of a ketogenic diet on asthma phenotypes, which can potentially complement disease management.

## 4. Materials and Methods

### 4.1. Reagents and Antibodies

A lung tissue dissociation kit (# 130-095-927) was purchased from Miltenyi Biotec (Gaithersburg, MD, USA). Freshly prepared Protein Extraction Buffer (PEB) buffer was reconstituted by adding 0.5% bovine serum albumin (BSA) to 1 X phosphate-buffered saline (PBS), pH 7.2, and 2 mM EDTA. The MACS BSA Stock Solution (# 130-091-376) was diluted at 1:20 with autoMACS^®^ Rinsing Solution (# 130-091-222) Miltenyi Biotec (Gaithersburg, MD, USA). Enzyme A and D were reconstituted by adding 1 mL and 3 mL of 1x buffer s to the lyophilized power respectively. gentleMACS c-tubes (# 130-096-334), MACS SmartStrainers (70 μm) (# 130-098-462), MACSmix™ Tube Rotator (# 130-090-753), and gentleMACS Dissociator (# 130-093-235) were all purchased from Miltenyi Biotec (Gaithersburg, MD, USA). Antibodies and compensation (# A10346) beads used for compensation were purchased from Bio-Legend (San Diego, CA, USA) and ThermoFisher (Waltham, MA, USA) The clone number and antibodies are provided in Table 1a–c. The conjugated fluorophores used in the study are enlisted in Table 2.

### 4.2. Animal Model

**Figure 10:**
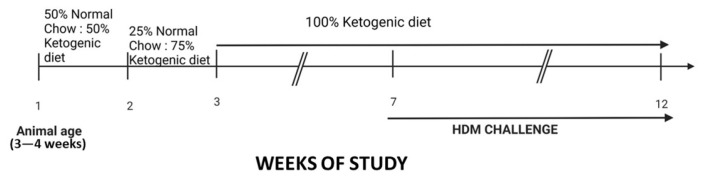
Male and female mice (*n* = 5–6 per group) were fed a ketogenic diet while the control animals were maintained on normal mice chow for 12 weeks from the age of 3–4 weeks old. These mice were challenged intranasally by administering 50 μL of House Dust Mite (HDM) solution (25 μg of HDM extract from two species, *Dermatophagoides pteronyssinus*, and *Dermatophagoides farinae*) at the 7th week of the experiment five times/week to the end of the 12-week study. At 72 h, animals were euthanized, and lungs were harvested and processed into single-cell suspension for flow analysis.

The animal study protocol (Protocol 23-008) was approved by Indiana University Bloomington’s Institutional Animal Care and Use Committee (BIACUC) on 08/10/2023 before the commencement of the study. Twenty-four Male and female (*n* = 10–12/group) C57BL/6J mice 3–4 weeks old were purchased from Jackson Laboratories (Bar Harbor, ME). After acclimating the mice, they were randomly separated into 4 groups of 6 males or 6 females per group to positive control and experimental groups: (1) male mice fed normal mice chow; (2) female mice fed normal mice chow; (3) male mice fed a ketogenic diet; and (4) female mice fed a ketogenic diet for 12 weeks (Figure 10). The sample size was determined based on our prior studies [27,28,29], based on the anticipated effect size in the immune response, specifically measuring of cell population percentage. Animals in the same group were housed in cages at Indiana University Bloomington animal facility and no animal was excluded from the study. To account for cage variance, animals in the same group were housed together and arranged in different columns on the same rack for the 12 weeks of study. This study was conducted in full compliance with the ARRIVE (Animal Research: Reporting of In Vivo Experiments) guidelines to ensure rigorous and transparent reporting.

### 4.3. Diet Protocol

The ketogenic diet (D15010301) and the control diet (D11112201) were obtained from ResearchDiets (New Brunswick, NJ, USA) while the ketogenic diet was stored frozen, the control diet was stored at room temperature. This diet contained 89% Fats (from both animals and plants), 10% Proteins, and 1% carbohydrates; the composition of the diet is shown in Table 3. The experimental male and female mice were maintained on the ketogenic diet, while the corresponding control groups had the normal mice chow throughout the experiment. Food in the cages was replaced twice each week. The different diets were commenced in the 1st week after acclimating the animals, and mice were maintained on this diet for 12 weeks of the study. The 12-week duration of the study has proven to be very sufficient to elicit changes in the mice, even in other animal studies [78,79,80].

### 4.4. HDM Challenge

The house dust mite (HDM) mix administration commenced in the 7th week of the 12-week study (Figure 10). Mice were administered 25 µg of the allergen extract from *Dermatophagoides pteronyssinus*, and *Dermatophagoides farinae* mix (Citeq Biologics, Groningen, The Netherlands) suspended in 50 µL of phosphate-buffered saline (PBS) daily (5 days/week) for a total of 5 weeks, intranasally, following a 5% isoflurane anesthesia using the SomnoSuite device (Kent Scientific, Torrington, CT, USA). This protocol induces a phenotypic allergic airway inflammation, which represents a physiologically relevant model to study mechanisms of airway inflammation in human asthma [29]. Also, the weight of the animals was measured at the end of the study using a scale, and the average value was compared among the groups.

### 4.5. Isolation of Immune Cells and Flow Cytometry

At 72 h after the last HDM challenge, mice from the different groups were first anesthetized using ketamine (100 mg/kg) + xylazine (10 mg/kg), each of the lungs was perfused by flushing 10 mL of cold PBS using a 10 mL syringe with a 25 G needle. The lung tissue was placed in the c-tube containing 2.4 mL of Buffer S, 15 μL of Enzyme A, and 100 μL of Enzyme D (Miltenyi Biotec, Gaithersburg, MD, USA # 130-095-927). The tube was then placed on the gentleMACS dissociator (Miltenyi Biotec, # 130-093-235) for homogenization. The homogenized tissue was incubated for 30 min at 37 °C and then placed on the tissue dissociator for the second time before passing the solution through the sterile SmartStrainers (70 μm, Miltenyi Biotec, Gaithersburg, MD, USA), rinsing with 2.5 mL of buffer S in a 15 mL tube. Cells were washed twice with PEB buffer and pelleted for all the experiments. The cells were then resuspended in PEB buffer and divided into individual tubes containing different antibodies depending on the cell type to be analyzed. The antibodies used to identify the 13 cell types were separated in 3 sets, as shown in Table 1a–c. Samples were incubated at room temperature for 30 min before washing with PEB and resuspending in 300 μL PEB buffer for flow cytometry analysis using a Beckman Coulter Life Sciences 4-laser (V-B-Y-R) CytoFLEX LX (Indianapolis, IN, USA) flow cytometer. At least 50,000 events were recorded for each analysis. The Zombie NIR (fixable viability dye) was used for live/dead cell discrimination. After collecting data, results were analyzed using the FlowJo software (FlowJo LLC, version 10.10 Ashland, OR, USA). The cells identified by the set 1 (Table 1a) antibodies included alveolar macrophages, eosinophils, CD103^+^ dendritic cells, CD11b^+^ dendritic cells, and interstitial macrophages. The cells identified by the set 2 antibodies included undifferentiated monocytes and neutrophils (Table 1b). The cells identified by the set 3 antibodies include plasmacytoid dendritic cells, B-cells, CD4^+^ T-cells, CD^+^8 T-cells, natural killer cells, and natural killer T-cells (Table 1c).

### 4.6. Gating Strategy

The gating strategy used for this study was adopted from previously published research [81]. Surface marker expressions used for immune cell strategies are included in Table 1a–c. All gating was based on live cell populations. Unstained cells and Fluorescence-Minus-One (FMO) controls were used to establish boundaries among immune cell populations. Single-color controls were used to develop proper compensation between fluorophores. Unstained, FMO, and compensation controls were run for each experiment and each set: set 1, set 2, and set 3. A representation of the gating strategy for each cell type investigated is shown in Supplementary Figure S1.

### 4.7. Statistical Analysis

Data were analyzed using two-way ANOVA on GraphPad Prism 10.2.1 with the treatment order blinded to ensure unbiased result. The interaction of diet and sex was determined for each variable, followed by post hoc Tukey’s test for multiple comparisons when relevant. Results were represented as graphs indicating each group’s Mean ± SEM. The *p* value of less than or equal to 0.05 was considered statistically significant. The description of the source of variation for each analysis is shown in the plotted graphs.

## 5. Conclusions

In conclusion, chronic ingestion of a ketogenic diet significantly altered the population of immune cells, including eosinophils, macrophages, dendritic cells, T-cells, and B-cells in male and female mice exposed to allergens. Most importantly, we observed that sex may play a role in these cell responses and, therefore, should be considered a major factor in diet and immunity research. Our data suggest that a ketogenic diet has been shown to alleviate inflammation in allergen exposure in a sex-specific manner and may complement the management of allergic airway inflammation, especially in males.

## Figures and Tables

**Table 1:** Antibodies, surface marker expressions, and clone numbers used to characterize the immune cells.

**a. Set 1**			
**Immune Cell Type**	**Antibody**	**Surface Marker Expression**	**Clone**
Alveolar macrophages	CD45-FITC	CD45+ Siglec F+ CD11b−	30-F11
Eosinophils	Siglec F-PE	CD45+ Siglec F+ CD11b+	S17007L
CD103+ DCs	CD11c-Percp/Cy5.5	CD45+ Siglec F− CD11b− CD103+ CD11c+ MHC II+	N418
CD11b+ DCs	CD11b-PE/Cy7	CD45+ Siglec F− CD11b hi CD103− CD64− MHC II+	M1/70
Interstitial macrophages	CD64-APC	CD45+ Siglec F− CD11b hi CD103− CD64+ MHC II+	X54-5/7.1
	CD103-BV421		2E7
	MHC II-BV510		M5/114.15.2
**b. Set 2**			
**Immune Cell Type**	**Antibody**	**Surface Marker Expression**	**Clone**
Monocytes/moDCs	CD45-FITC	CD45+ CD11b hi Ly6C hi/int CCR2+/− Ly6G−	30-F11
Neutrophils	Ly6C-PE	CD45+ CD11b hi Ly6C int CCR2− Ly6G+	HK1.4
	CD11b-PE/Cy7		M1/70
	CCR2-BV421		SA203G11
	Ly6G-BV510		1A8
**c. Set 3**			
**Immune Cell Type**	**Antibody**	**Surface Marker Expression**	**Clone**
Plasmacytoid DCs	CD45-FITC	CD45+ B220+ CD11c+	30-F11
B-cells	CD8-PE	CD45+ B220+ CD11c−	53-6.7
CD4+ T-cells	NK1.1-Percp/Cy5.5	CD45+ B220− CD11c− CD4+ CD8−	PK136
CD8+ T-cells	CD11c-PE/Cy7	CD45+ B220− CD11c− CD4− CD8+	N418
NK cells	APC-B220	CD45+ B220− CD11c− CD4− CD8− NK1.1+ TCRb−	RA3-6B2
NKT cells	CD4-BV421	CD45+ B220− CD11c− CD4− CD8− NK1.1+ TCRb+	GK1.5
	TCRb-BV510		H57-597

**Table 2:** Antibodies, conjugated fluorophore, CytoFLEX detector and quantity of antibodies used to analyze the immune cells.

Target	Fluorochrome	CytoFLEX LX Laser/Detector	Quantity
B220/CD45R	APC	640 nm/R660	0.25 µg per 10^6^ cells in 100 µL volume
CCR2	BV421	405 nm/V450	0.5 µg per million cells in 100 µL volume
CD103	BV421	405 nm/V450	5 µL per million cells in 100 µL staining volume
CD11b	PE/Cy7	561 nm/Y763	0.25 µg per 10^6^ cells in 100 µL volume
CD11c	PE/Cy7	561 nm/Y763	0.25 µg per 10^6^ cells in 100 µL volume
CD11c	PerCP/Cyanine5.5	488 nm/B690	1.0 µg per million cells in 100 µL volume
CD4	BV421	405 nm/V450	5 µL per million cells in 100 µl
CD45	FITC	488 nm/B525	0.25 µg per 10^6^ cells in 100 µL volume
CD64	APC	640 nm/R660	1.0 µg per million cells in 100 µL volume
CD8	PE	561 nm/Y585	0.25 µg per 10^6^ cells in 100 µL volume
Ly6c	PE	561 nm/Y585	0.25 µg per 10^6^ cells in 100 µL
Ly6g	BV510	405 nm/V525	0.5 µg per million cells in 100 µL volume
MHCII	BV510	405 nm/V525	5 µL per million cells in 100uL staining volume
Nk1.1	PerCP/Cyanine5.5	488 nm/B690	1.0 µg per million cells in 100 µL volume
Siglec F	PE	561 nm/Y585	0.25 µg per million cells in 100 µL volume
TCRb	BV510	405 nm/V525	5 µL per million cells in 100 µL staining volume
Dead cell stain	Zombie NIR	640 nm/R763	1:100 for 1–10 million cells
TruStain FcX™ (anti-mouse CD16/32) Antibody	N/A	N/A	1.0 µg per 10^6^ cells in 100 µL volume

**Table 3:** Composition of research diets.

Product	Control Diet (D11112201)		Ketogenic Diet (D15010301)	
	gm%	kcal%	gm%	kcal%
Protein	19	20	17	10
Carbohydrate	63	65	2	1
Fat	7	15	66	89
Total		100		100
kcal/gm	3.81		6.6	
Ingredient	gm	kcal	gm	kcal
Casein	200	800	100	400
L-Cystine	3	12	3	12
Corn Starch	381	1524	0	0
Maltodextrin 10	110	440	0	0
Sucrose	150	600	0	0
Cellulose, BW200	75	0	50	0
Inulin	25	37.5	0	0
Soybean Oil	70	630	25	225
Lard	0	0	175.5	1580
Cocoa Butter	0	0	200	1800
Mineral Mix, S10026	10	0	10	0
Dicalcium Phosphate	13	0	13	0
Calcium Carbonate	5.5	0	5.5	0
Potassium Citrate, 1 H2O	16.5	0	16.5	0
Vitamin Mix, V10001	10	40	10	40
Choline Bitartrate	2	0	2	0
FD&C Yellow Dye #5	0.025	0	0.025	0
FD&C Red Dye #40	0	0	0.025	0
FD&C Blue Dye #1	0.025	0	0	0
Total	1071.05	4084	610.55	4057

## Data Availability

All sources of data can be found using the following link: https://figshare.com/s/af9c32607e01017b46fe, accessed on 2 March 2025.

## Supplementary Materials

The following supporting information can be downloaded at: https://www.mdpi.com/article/10.3390/ijms26073046/s1.
