# Supplementary material for: Sex-Specific Anti-Inflammatory Effects of a Ketogenic Diet in a Mouse Model of Allergic Airway Inflammation
Source: Int J Mol Sci. 2025 Mar 26;26(7):3046. doi: 10.3390/ijms26073046 (PMC11989016; doi:10.3390/ijms26073046)
Supplement: Supplementary file 1 [file ijms-26-03046-s001.zip › ijms-3536891-supplementary (for conversion) v3.pdf]

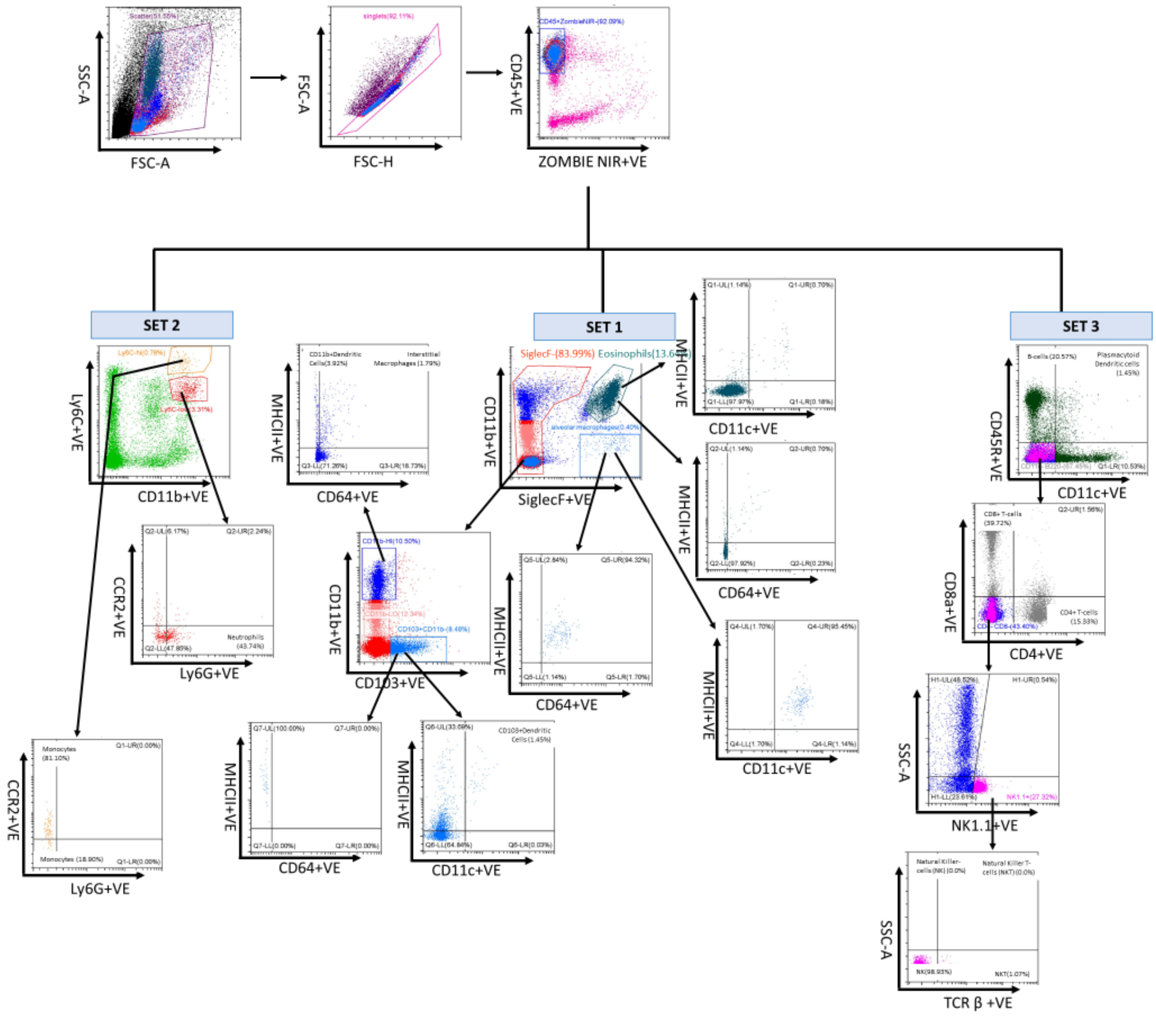

Supplementary Figure 1: Lung tissues were excised 72 hrs after the 12-week study, and 13 immune cells were analyzed using flow cytometry, defined by the surface markers listed in Table 1a–c. For set 1 antibodies, we gated CD45^{+} viable cells on Siglec F and CD11b to mark out CD11c and MHC II expressing alveolar macrophages and eosinophil populations, which do not express CD11c and MHC II. Cells within the Siglec F population were then gated to identify CD103 and CD11b^{+}. CD103^{+} CD11b- expressions. CD11c and MHC II-expressing cells and not CD64 were further gated, CD103^{+} dendritic cells. The CD11b high population was gated to identify cells that express CD64 and MHC II. CD11b^{+} dendritic cells did not express CD64 but MHC II, while the cells identified as interstitial macrophages express both CD64 and MHC II. For set 2 antibodies, CD45^{+} viable cells were gated to determine the CD11b and Ly6C expressing population. Undifferentiated monocytes were identified from Ly6C high cells that express CCR2 while neutrophils were identified from Ly6C low population. Also, for set 3 antibodies, CD45 $^{+}$ viable cells were gated to identify B-cells and plasmacytoid dendritic cells expressing CD11c and CD45R. CD4^{+} and CD8^{+} cells were identified by gating CD11c^{-} and CD45^{-} to determine CD4 and CD8-expressing cells. Cells not expressing CD4 and CD8 were further gated to determine NK1.1- and TCRβ-expressing ones, which were labeled natural killer cells and natural killer T-cells.
